# Cold Central Plant Recycling Mixtures for High-Volume Pavements: Material Design, Performance, and Design Implications

**DOI:** 10.3390/ma18143345

**Published:** 2025-07-16

**Authors:** Abhary Eleyedath, Ayman Ali, Yusuf Mehta

**Affiliations:** Center for Research and Education in Advanced Transportation Engineering Systems (CREATES), Rowan University, Glassboro, NJ 08028, USA; alia@rowan.edu (A.A.); mehta@rowan.edu (Y.M.)

**Keywords:** cold central plant recycling, recycled asphalt pavement, recycling agents, foamed asphalt, emulsified asphalt

## Abstract

The cold recycling (CR) technique is gaining traction, with an increasing demand for sustainable pavement construction practices. Cold in-place recycling (CIR) and cold central plant recycling (CCPR) are two strategies under the umbrella of cold recycling. These techniques use reclaimed asphalt pavement (RAP) to rehabilitate pavement, and CCPR offers the added advantage of utilizing stockpiled RAP. While many agencies have expertise in cold recycling techniques including CCPR, the lack of pavement performance data prevented the largescale implementation of these technologies. Recent studies in high-traffic volume applications demonstrate that CCPR technology can be implemented on the entire road network across all traffic levels. This reignited interest in the widespread implementation of CCPR. Therefore, the purpose of this study is to provide agencies with the most up-to-date information on CCPR to help them make informed decisions. To this end, this paper comprehensively reviews the mix-design for CCPR, the structural design of pavements containing CCPR layers, best construction practices, and the agency experience in using this technology on high-traffic volume roads to provide in-depth information on the steps to follow from project selection to field implementation. The findings specify the suitable laboratory curing conditions to achieve the optimum mix design and specimen preparation procedures to accurately capture the material properties. Additionally, this review synthesizes existing quantitative data from previous studies, providing context for the comparison of findings, where applicable. The empirical and mechanistic–empirical design inputs, along with the limitations of AASHTOWare Pavement ME software for analyzing this non-conventional material, are also presented.

## 1. Introduction

Pavement recycling is the process of reusing the milled-off deteriorated pavement materials back into the pavement structure for rehabilitating an existing pavement or constructing a new pavement. It reduces pavement construction costs [1,2] by reducing the requirement of virgin binders or aggregates. Recycling also helps to reduce the negative environmental impacts caused by the accumulation of reclaimed pavement materials as landfill waste and reduce greenhouse gas emissions [1,2,3]. The utilization of existing pavement also reduces truck haul times and makes the construction process quick and efficient [1,2,3]. There are three most used pavement recycling processes: hot recycling (HR), full-depth reclamation (FDR), and cold recycling (CR) [4]. In hot recycling, 40% of reclaimed asphalt pavement (RAP) is allowed in base layers, while in cold recycling 100% RAP is permitted [5,6]. The increasing popularity of cold recycling can be attributed to its cost-effectiveness and environmental benefits. Compared to conventional asphalt technology, cold recycling reduces lifecycle costs by 30% to 50% [7]. Using RAP allows the USA to save more than 3.3 billion dollars by reducing the use of natural resources like virgin aggregates [8]. Additionally, this technology reduces fuel consumption and greenhouse gas emissions [2,8,9].

### 1.1. Issues with Stockpiled RAP and Potential Solutions

**Figure 1:**
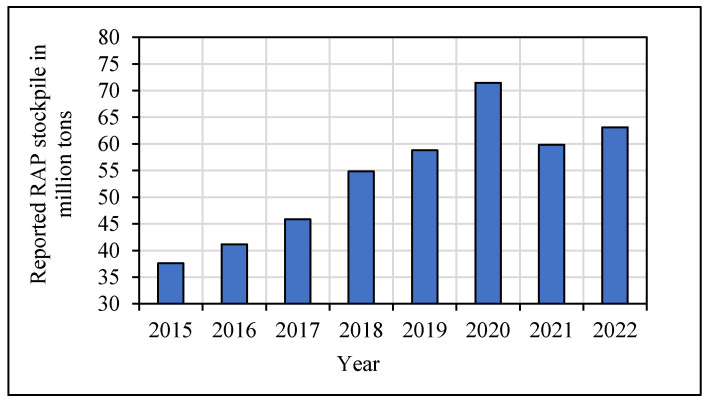
Variation in RAP stockpile in USA.

Recent studies show that RAP stockpiling is rising in the United States [10,11]. This increasing trend can be observed from the National Asphalt Pavement Association (NAPA) survey results [12,13,14,15] as shown in Figure 1. 

The trend changes after 2020 might be due to the increased utilization of stockpiled RAP in the pavement construction and the increased availability of RAP stockpile inventory. The quantity of stockpiled RAP reported by NAPA is significantly underestimated, as indicated by a recent study conducted in Washington using Google Earth Pro. Between 2006 and 2017, the RAP stockpile rose by 190% [16]. Due to its variability, stockpiled RAP is often disposed of in landfills. The accumulation of this waste over time eventually becomes an environmental burden, and agencies and industries face difficulties disposing of it. Cold central plant recycling (CCPR) technology utilizes stockpiled RAP to place new, stabilized base layers in pavements. Williams et al. [13] estimated that 68 million cubic yards of landfills can be cleared by reusing the stockpiled RAP. In addition, unused asphalt mixtures from manufacturing facilities can also be incorporated into CCPR material to prevent wastage [17]. As a result, more agencies are utilizing this technology, and cold central plant recycling is becoming popular.

### 1.2. Overview of CCPR

CCPR is a process of incorporating RAP from highway millings or existing RAP stockpiles as bound (or stabilized) base layers into newly constructed or reconstructed pavements [18,19]. CCPR was used as an unbound base course [20] and the sub-base course [5]. The technology involves mixing RAP materials with emulsified or foamed asphalt (binding or recycling agent) in a central (stationary or mobile) facility at ambient temperatures. The preprocessing of RAP materials, such as screening, crushing, and sizing, will enhance CCPR material quality. To aid in mixing, water is added (1 to 3%) [21]. Water contents of up to 5% are allowed in certain conditions. In the absence of proctor test results, foam-stabilized CCPR mixes are generally assumed to have an optimal moisture content of 5%. Active filters/chemical additives are also used to enhance the CCPR mixture. Cement is the most used filler, followed by lime, fly ash, and lime kiln dust (often 1%). The CCPR mixture is transported to the construction site and placed as a stabilized base layer using conventional equipment.

### 1.3. Advantages of CCPR

CCPR offers several advantages, including reusing enormous quantities of RAP materials in a cost-effective and environmentally responsible manner, conserving aggregate natural resources by using readily available RAP materials, and upgrading existing pavements without altering their structure. The CCPR technique allows more control over RAP gradation and recycles thicker pavement sections than CIR [16,21,22]. In general, CCPR projects saved 54% in cost compared to HMA applications [21].

Managing the recycled RAP material gradation differs between CIR and CCPR techniques. CIR relies on the speed of the reclaimer, whereas CCPR employs mechanical sieving [16]. Even though the two technologies, namely the CIR and CCPR, have similar characteristics, there are some specific advantages for CCPR [21]. CCPR is useful when CIR is not feasible due to logistical reasons or when underlying layers need additional treatment. CCPR allows more control over recycling and the exploitation of RAP from various sources [5,22]. This enables greater pavement layer thicknesses and design life to be achieved with CCPR compared to CIR [9,23]. A thicker pavement will resist moisture-damage and eventually improve the pavement’s resilience to flooding [24,25,26]. Hence, CCPR can fix structural problems when used as a foundation layer. Unlike traditional pavement recycling practices (asphalt concrete milling and repaving), CCPR technology addresses the root cause of pavement structural distress rather than curing the symptoms [16]. It reduces the negative environmental impact by approximately 16% [27]. CCPR can use both millings from the reclamation process and stockpiled RAP, allowing its use for new construction and rehabilitation of pavements [2].

While cold central plant recycling (CCPR) offers significant potential for sustainable pavement rehabilitation, several scientific challenges remain in optimizing its widespread implementation. These challenges include variability in reclaimed asphalt pavement (RAP) materials, which affects the consistency of mix design and the performance of CCPR mixtures. Additionally, traditional pavement design models are often insufficient to predict the long-term performance of CCPR pavements, as these models typically focus on conventional HMA mixtures. The long-term durability, fatigue resistance, and environmental sustainability of CCPR pavements are also areas that require further research to understand how these materials perform under various traffic and climatic conditions. Finally, the curing behavior of CCPR mixtures and their temperature sensitivity during construction remain critical factors that need to be addressed to ensure consistent pavement quality. These scientific challenges, which are discussed in detail throughout this article, highlight the need for further research to optimize CCPR technologies and enhance their application in diverse pavement structures.

### 1.4. Laboratory Performance of Laboratory-Mixed and Field CCPR Specimens

In contrast to CIR mixtures, CCPR mixtures have lower gradation variability [16,19]. This is due to the controlled preprocessing of RAP in the central plant. The density gradient between the top and bottom CCPR cores [16] is comparable to that observed in conventional mixtures after compaction. The density of the in-place mix and lab mix also varies [4,28]. Some studies demonstrated that CCPR mixture, like conventional asphalt concrete, is stiffer at lower temperatures [16]. Similar observations were made with lower air voids, and the CCPR mixture was less stiff at higher air voids than the conventional asphalt concrete. The compaction method and air voids affect the stiffness of the CCPR mixture but not the permanent deformation properties. CCPR mixtures withstand permanent deformation and temperature cracking better than conventional mixes.

A limited study on field-cored specimens showed that the CCPR mixture had better fatigue resistance than traditional mixtures [29]. Due to rheological changes, dynamic modulus prediction models for asphalt concrete mixes are not applicable to CCPR mixtures [5,21]. Laboratory-determined dynamic modulus values are available for CCPR mixtures that serve as an input into the Pavement ME Design software version 3.0 [30]. However, fatigue and rutting results are limited [21]. The field core and lab specimen of emulsion-stabilized CCPR mix satisfied the tensile strength ratio requirement of 75% [31]. The field core from a rehabilitation project with emulsion-stabilized CCPR mix (100% RAP, 3.5% emulsion, 1.5% cement) showed improvement in resilient modulus, rut resistance, and moisture susceptibility after 12 months of construction [32].

### 1.5. Field Implementation of CCPR

**Figure 2:**
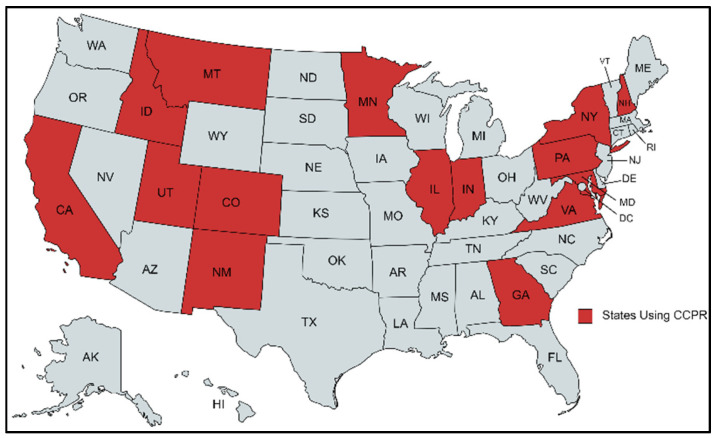
States employing CCPR construction practice in USA [21]. Source: https://www.mapchart.net/usa.html (accessed on 20 May 2025).

CCPR usage appears to be substantially lower than CIR [23] in the United States and Canada [32], European countries [5], and Asian countries [29,32,33]. CIR technology has been examined extensively in comprehensive reviews of cold recycling methods [7,23,28,34]. Agencies have adopted CIR extensively in the USA [8,9,35,36,37], but CCPR has not been widely used in rehabilitation. The use of CCPR on high-traffic roads is limited due to the lack of performance history data. Figure 2 shows the states that have adopted CCPR in pavement construction.

As the first agency to use CCPR in high-volume road construction, VDOT conducted an experiment in Virginia that found CCPR mixtures to perform comparably to conventional asphalt concrete mixtures [38,39]. In high-traffic volume applications, studies propose CCPR mixtures with ITS > 310 kPa and a tensile strength ratio > 70% [40]. VDOT, MNDOT and NCAT test sections are presented in detail along with the discussion on structural design. CCPR mix with 75% RAP, 25% virgin aggregate, 2.5% foam bitumen, and 1% cement was used to improve the moisture damage resistance of an Indian road [22]. A Malaysian road rehabilitated using an emulsion-stabilized CCPR mix performed well without any significant rutting or fatigue failure after 12 months of construction [32]. Germany’s high-traffic road A555 was reconstructed with a CCPR mix composed of 75% RAP, 25% crushed sand, 2% foam bitumen, and 1% cement in 2024 [31,41] and the performance data is not available for the test sections constructed in 2020 [28]. This review synthesizes existing studies on pavement sections incorporating CCPR layers. Detailed analyses of these sections, including the methodologies used, are found in the cited literature. Even with significant benefits, CCPR technology is not widely adopted by agencies due to the lack of unified guidelines for mix-design, structural design, and construction practices. There is also limited data on the performance history and an ambiguity on the properties needed for the structural design of CCPR. Hence, the overall goal of this study is to provide a comprehensive, unified overview of the current state of knowledge regarding the pavements containing CCPR layers, based on the available literature. The following set of specific objectives is defined:

Evaluate the mixture design procedures for CCPR and its impact on structural design.

Identify the most suitable Level 1 input parameters for the Pavement ME analysis of pavements that contain CCPR layers.

Discuss the best construction practices for CCPR layers for high-traffic roads.

## 2. Selection Criteria for CCPR Projects

The criteria for the selection of the CCPR project depends on the following factors:

*Size of the project:* CCPR mixtures can be applied as an unbound base course or a bound base course in pavements of any length [7,8]. As the technology is using stockpiled RAP material and the mixing is carried out in a mobile plant, the project length will not be a constraint.

*Roadway classification*: CCPR technology can be implemented across all traffic levels. Previously this technology was limited to low and medium traffic roadways. However, recent studies proved that CCPR can be effectively adopted in high-traffic roadways as well [3,4,7,22,29,31,32].

*Type of project:* Both the new construction and rehabilitation projects are suitable candidates for applying CCPR technology [7,22,27,38,42].

*Roadway geometry:* Roadway geometrics will not impede the application of CCPR, and as a result, it is suitable for use in both urban and rural areas [22,42]. The maneuverability of the long CIR train will be a problem in urban areas [22]. However, CCPR allows the use of small recycling units [22,42].

*Pavement distress evaluation:* Detailed pavement distress evaluation data will help to decide the feasibility of CCPR application. A pavement with surface cracking, non-load related distress and roughness will be a good candidate for CCPR. The pavement with deep structural failures can also be selected after stabilizing or replacing the damaged foundation layers [42]. CCPR can be used when the subgrade soil, base, and subbase layers are sufficiently resilient, and only the surface layers are damaged [9,21]. The I-81 pavement was constructed in 1967-68, and a 2006-07 evaluation found load-induced cracking, pumping, and stripping [4,10,20,43,44]. VDOT chose the reconstruction of I-81 pavement with deep structural failures as a candidate structure for CCPR technology [4,16,43,44].

*Drainage condition:* The site selected to apply CCPR technology required to have adequate drainage facility [7,22,42]. However, a recent study in Malaysia used CCPR on a pavement located at the wettest area with poor drainage and the construction happened during light rain. The performance history of this project will reveal the moisture susceptibility of the CCPR [32].

The field investigations, testing of field cores, and adequate subgrade support are also essential to project selection [21].

## 3. Materials Used in CCPR Mixtures

### 3.1. Reclaimed Asphalt Pavement (RAP)

RAP generated by cold milling of existing asphalt pavements on roads within work limitations [14,45] or the stockpiled RAP can be used for CCPR. Crushed and screened RAP, without dirt, foundation material, concrete, or other detrimental substances is required for CCPR [46,47] as given in Table 1. The gradation requirements are given in Table 2.

### 3.2. Recycling Agent for CCPR

Foamed asphalt or emulsified asphalt are the two recycling agents used in the process of CCPR. Typically, the binder that can be foamed is suitable for the CCPR mixture [37,47]. Although softer binders often have better-foaming properties than stiffer binders, foamability cannot be judged from the binder grade. Some crude sources do not produce binders that foam well. A binder with an anti-foaming agent or polymer-modified binders will not foam, regardless of its performance grade. The binder grade for CCPR is usually the same as that used in surface HMA. The binder temperature between 160 and 190 °F will be good to achieve adequate foaming with a minimum expansion ratio of 11 and a half-life of 8 s [7,22,42].

The research conducted by VDOT for the I-81 project used PG 64-22 asphalt binder [16], and for the two sections built in the NCAT test track [50] used PG 67-22 in the CCPR layer. The 50/70 Styrene Butadiene Styrene modified binder with a penetration value of 60 dmm was used in the study conducted on the Italian motorway [5]. Gu et al. [51] used PG 67-22 and PG 64-22 binders for the foaming process in the CCPR mixture design. The test sections constructed in 2017 at MnROAD used PG 58S-28 and Minnesota PG XX-34 binders [39]. VDOT used 2% foamed asphalt to produce the CCPR mixture for I-81 [16,52]. India used south African Guideline TG2,2009 for the foam-stabilized CCPR project [22].

Emulsion treatment introduces extra moisture into the mix and requires extended curing before the road can be opened to traffic. CSS-1h, SS-1h, HFE 150 or engineered emulsions are suitable for use in CCPR mix. Emulsion produced from 60/70 grade binder was used in a project as per Chinese guidelines [32]. AASHTO PP-86 [53] provides guidelines for the use of emulsion in CCPR mixes. Use the emulsion within the storage life recommended by the supplier, avoid heating it, and ensure that it is free from separation.

These studies indicate the following requirements for the recycling agents in CCPR mix:

A binder that can be foamed is suitable for the foam-stabilized CCPR mix.

Minimum expansion ratio of 11 and a half-life of 8 s is required for foamed CCPR mix.

Avoid using a binder with an anti-foaming agent or modifying agent.

Use an engineered emulsion for the emulsion-stabilized CCPR mix.

Follow suppliers’ recommendations for the storage and handling of the emulsion.

### 3.3. Chemical Additive

The chemical additives used are hydraulic cement, lime, lime kiln dust, and fly ash. VDOT used 1% cement in NCAT test sections and I-81 recycling project [16,52]. Cement is used to increase the #200 material to help distribute foam bubbles throughout the mixing matrix [8,15,42], and to enhance the moisture resistance of the mixture [2,5,16]. The cement content in the CCPR mixture contributed to the resistance to permanent deformation, adversely affecting fatigue resistance [5]. Lime or cement (at 1 to 2% by weight of dry RAP) was added as a recycling additive to make the CCPR mixture stiffer and more resistant to moisture [39]. A ratio of at least 3:1 between residual asphalt in the bituminous recycling agent and dry cement is also required [13]. In general, 1% of cement is used in foam-stabilized CCPR mixes and 1 to 1.5% cement in cationic emulsion-stabilized CCPR mixes [32,54]. Cement is not used as an additive in anionic emulsions due to their alkaline nature [32,54].

## 4. CCPR Mixture Design

General mixture design establishes the type of materials used, their percentage, and the optimal binder content. The high variability associated with RAP material prevents field adjustments after placing the CCPR mixture. Using samples from stockpiles, the chemical and physical characteristics of the RAP binder are investigated. The stockpile materials are crushed to make recycled RAP, which is further processed to meet AASHTO T 27 gradation specifications. Identifying the type and grade of recycling agent to be added to the mixture is the next step in design. Emulsified asphalt or foamed asphalt is used as a recycling agent.

After mixing and curing, the specimen may be evaluated for Marshall Stability, Indirect Tensile Strength (ITS), and low-temperature creep strength. Specimens are soaked to 50–60% for Marshall Stability and ITS. Per ARRA and AASHTO guidelines [39], the CCPR mix design specimens are compacted with 30 gyrations using a Superpave gyratory compactor. The effect of curing time on the performance of the CCPR mixture needs further research [20]. The requirements for the foam-stabilized and emulsion-stabilized CCPR mixtures are provided in Table 3 and further details are available in the literature [22,42].

## 5. Structural Design of Pavements Containing CCPR Layers

The current version of AASHTOWare software version 3.0 based on the mechanistic–empirical pavement design method does not provide options for analyzing pavements with asphalt-stabilized CCPR layers. Moreover, the distress prediction models developed for dense-graded Hot Mix Asphalt (HMA) are not suitable for cold mixtures [11,61]. Further, there is a lack of local calibration coefficients for the long-term performance of cold recycled mixtures [51]. Thus, studies that explored CCPR technology preferred the AASHTO 93 [62] method to design the CCPR layer as an asphalt-stabilized base layer. The discussion of various pavement sections with CCPR layer is a summary of findings from multiple studies. The referenced literature, cited in this review, provides further details on the methodologies employed for these analyses.

AASHTO 93 design optimizes the layer thickness based on the structural layer coefficient. Researchers reported that the characteristics of the CCPR layer are like that of the granular base material [40,50], AC layer [23] and vary between that of a granular base, and a conventional asphalt layer [63,64]. Studies using AASHTO 1993 pavement design method [62] for CCPR construction employ a range of structural layer coefficients that typically vary between the granular base values and conventional asphalt concrete. The reported values of the structural layer coefficient for the CCPR layer from different studies are presented in Table 4.

The low values for structural layer coefficient come from states that either evaluated old pavements from the 1970s–1990s that used cutback emulsions or used a “prescriptive” design, meaning those states did not do a mix design per their specifications [21,65,66]. Higher values are typically from mixtures with an actual mix design performed [21,50]. Researchers have modified pavement structure design guidelines based on the AASHTO 93 method to accommodate CCPR layers [50]. The pavement structures containing the CCPR layer designed and constructed by the Virginia Department of Transportation (VDOT), the National Centre for Asphalt Technology (NCAT), and the Minnesota Department of Transportation (MnDOT) are summarized below to understand the structural design aspects better.

**Figure 3:**
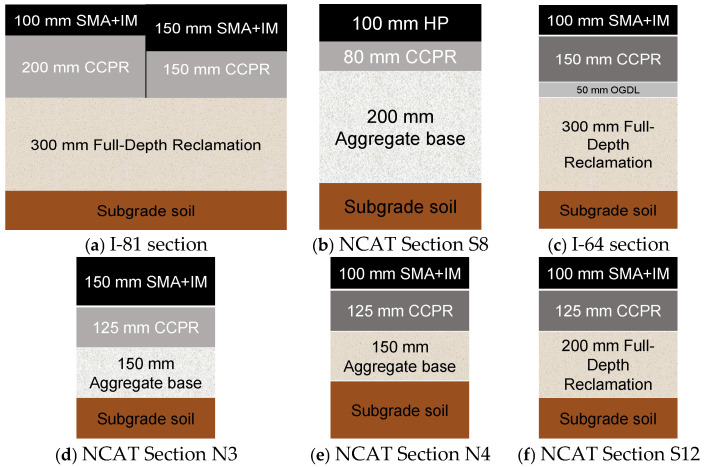
VDOT pavement sections with CCPR layers.

VDOT has been actively contributing to the advancement of cold recycling technologies, particularly CCPR. VDOT’s structural layer coefficient for CCPR is 0.35, based on the AASHTO 1993 approach. When CCPR is included in the pavement structure developed by the MEPDG approach, it is designed as a VDOT Base Mix with an increased thickness of 1.26 times [52,67]. The sections of the I-81 highway restored by VDOT, and the NCAT test track sections can be seen in Figure 3. The combined layer coefficient for the CCPR and FDR layers was 0.37, and the individual layer coefficient for the CCPR layer was 0.37–0.44 [52]. Section N3 had an average layer coefficient of 0.39 for the CCPR layer, while Section N4 had an average of 0.36. The coefficient for Section S12 was not computed [37].

**Figure 4:**
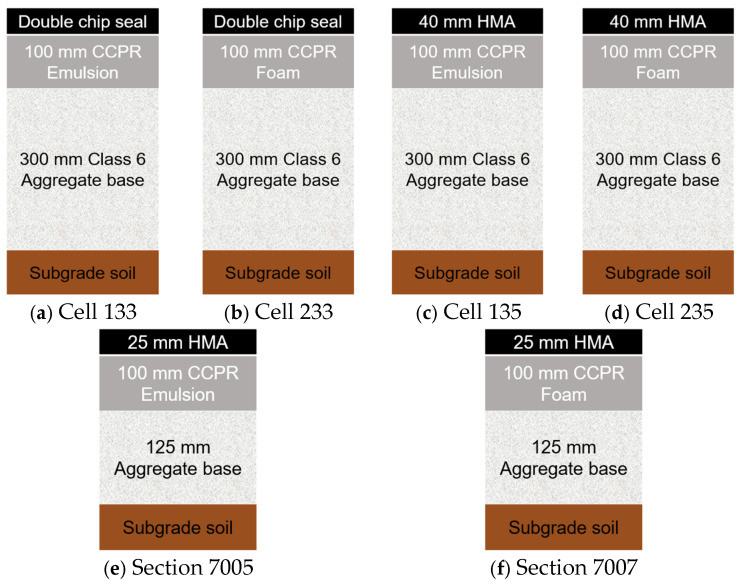
MnDOT CCPR sections.

MnROAD low-volume test track sections and the rehabilitated sections of 70th street roadway are illustrated in Figure 4 [21,39]. Based on the traffic information available for the pavement structures in Figure 3 and Figure 4, I-81 experiences 8000 trucks per day, and the CCPR pavement on this section has carried more than 20 million ESALs. Similarly, NCAT Sections N4 and S12 sustained over 27 million ESALs by 2021, while Section N3 was loaded with 20 million ESALs, demonstrating the effectiveness of CCPR under heavy traffic conditions. Additionally, I-64 carries approximately 3200 trucks per day, further showcasing the high-volume nature of these roadways. In contrast, the 70th Street roadway has an Average Annual Daily Traffic (AADT) of 2300 vehicles per day, reflecting a much lower traffic volume. These data points highlight the performance of CCPR in varying traffic conditions and provide context for its application in both high- and low-traffic areas. However, a definitive interpretation of the traffic levels and corresponding pavement structure would require further research.

The layer coefficient of CCPR in the New Hampshire study was 0.28 [68,69]. The Pennsylvania Department of Transportation merged CCPR and FDR, using 0.37 as the layer coefficient. The Indiana Department of Transportation (INDOT) rehabilitated State Route 101, a major collector route for US-24, in the Fort Wayne District with CCPR technology [70,71,72]. CCPR technology was used to reconstruct the road stretch from SR 145 to Fig Avenue in Fresno, California [73,74].

Recent studies showed that pavements with CCPR layers atop an FDR layer behave more like perpetual pavements than conventional pavements based on the empirical AASHTO 93 approach [2,3,75]. The probabilistic nature and mechanistic–empirical design methodology of perpetual pavement construction characterizes its design. Instead of prescribing a single number for the limiting strain, the perpetual design proposes a distribution of limiting strain values [76]. Designs for perpetual pavements have a 50-year design life and are intended for long-lasting pavements [77]. This design focuses on the pavement’s resistance to bottom-up fatigue cracking since fatigue failure is the leading cause of pavement deterioration, which requires expensive repair [12,25,77,78]. CCPR will need a new strain distribution distinct from those specified for traditional dense graded hot mix asphalt pavements [75]. Nonetheless, this finding will encourage the usage of perpetual pavement designs for high-traffic volume highways built using CCPR layers.

## 6. Field Performance of Pavements Containing CCPR Layers

Both sections of I-81 in Figure 3a demonstrated excellent riding quality after 34 months, with the section with the 8 in (200 mm) CCPR layer having a lower rut depth and a higher IRI value than the section with the 6 in (150 mm) CCPR. The pavement section with CCPR layer over the cement-stabilized FDR layer in Figure 3b was found to be less susceptible to temperature effects, stiffer, and exhibited better structural performance compared to the pavement section with CCPR layer over the conventional aggregate base [50,52]. The construction of the CCPR section on I-64, as shown in Figure 3c, was completed by 2021, and performance data is unavailable [79]. The results from the NCAT study (Figure 3d–f) showed that new pavements could be constructed with a high percentage (80%) of recycled content without compromising structural performance [50]. The N3 section with a 6 in (150 mm) overlay was dropped from further analysis, and the subsequent study found perpetual behavior for the S12 section [80]. MnDOT study demonstrated that CCPR with a 1.5 in (40 mm) HMA overlay (Figure 4) performed like a conventional section. Pavement ME overestimated rut depth in a CCPR study using bituminous recycling agents [51].

This review demonstrates that cold central plant recycling (CCPR) as a base layer qualifies as a high-value utilization of reclaimed asphalt pavement (RAP), especially in high-traffic conditions. By reusing 100% RAP in the base layer, CCPR reduces the need for virgin materials, such as new asphalt binder and aggregates, promoting sustainability and resource conservation. This method not only lowers material costs but also reduces the carbon footprint associated with the production of new asphalt [22,81]. Moreover, CCPR contributes to the circular economy by minimizing waste and maximizing the reuse of materials. As modern pavement engineering increasingly prioritizes sustainable infrastructure, CCPR aligns with these goals by providing a cost-effective, environmentally friendly, and durable solution for pavement rehabilitation. The benefits of CCPR make it a highly relevant technique for the efficient and sustainable recycling of RAP, addressing the evolving needs of infrastructure [31].

## 7. Summary of Findings and Conclusions

The following key findings are drawn from the synthesis of existing literature:

**Mixture Design:**

RAP materials smaller than the 1.5-inch size are used in the CCPR mixtures.

CCPR mixtures are compacted in the Superpave gyratory compactor with 30 gyrations.

The presence of cement will increase the moisture resistance of the mixture, and more than 1% cement will lead to an increase in brittleness.

The viscous component in CCPR mixes with cement is reduced at elevated temperatures.

CCPR mixes containing bituminous recycling agents (0.5 to 4% emulsion and 0.5 to 3% foamed asphalt) had viscoelastic behavior similar to that of conventional mixtures.

The prediction models primarily designed for traditional asphalt mixes cannot be used for CCPR mixtures.

**Structural Design of High Traffic Volume Pavements with CCPR Layers:**

Due to its simplicity, the AASHTO 93 guideline based on the traditional empirical design approach is commonly used in studies to design pavements with CCPR layers.

The structural layer coefficients vary between 0.36 and 0.44, while the CCPR layer’s thickness varies between 3 and 6 in (75 and 150 mm) in a single lift.

The CCPR layer, combined with a 4 in (100 mm) HMA overlay and a full-depth reclamation layer, outperformed conventional pavement construction.

Thicker pavement sections are possible with multiple lifts of CCPR layers that make it a perpetual pavement. Perpetual design for pavements with CCPR layers reduces construction costs and extends pavement life.

**Construction Practices:**

The RAP gradation requirement is an essential criterion for the CCPR mixture.

Due to its better control over mixture design, CCPR technology allows the pavement structure to preserve the correct geometry (profile and cross-slope).

CCPR using foamed asphalt cures faster than when using emulsified asphalt.

## 8. Recommendations

**Mixture Design:**

A binder that meets the foaming requirements is recommended for CCPR mixtures.

If the processed RAP does not meet gradation criteria, a corrective/new aggregate may be added.

Future research should evaluate the HMA performance prediction models to determine if they also apply to CCPR mixtures and modify them if required.

**Structural Design of High Traffic Volume Pavements with CCPR Layers:**

Pavements containing CCPR mixtures require a different limiting strain distribution than dense-graded HMA pavements for their perpetual design.

The performance history of pavements with CCPR layers must be documented to determine the long-term performance.

The fatigue characteristics of the CCPR mixture must be studied in detail to determine if the fatigue cracking concept applies to CCPR mixtures as to HMA mixtures.

**Construction Practices:**

Weak underlying layers need to be repaired before placing the CCPR layer.

A minimum ambient temperature of 50 °F is recommended for the placement of CCPR layers.

The paver speed required to be reduced by about 50% (13 ft/min) to place the CCPR mix, as opposed to the placement of the hot mix.

Single-lift thickness of 3 to 6 inches is recommended, and multiple lifts can be used to achieve higher thickness. Vibratory, rubber tire rollers, pneumatic tire rollers or steel drum rollers can be utilized for the compaction.

Nuclear gauge can be used to verify or accept the target density.

A minimum 3-day curing period is stipulated for the CCPR layer before placing an overlay.

It is recommended to avoid secondary compaction when using CCPR mixture with cement.

On the CCPR layer, rolling traffic is permitted after 2 h of fog sealing.

A tack coat is required on top of the CCPR layer prior to the installation of the surface course; however, a heated asphalt binder should not be employed.

## Figures and Tables

**Table 1:** RAP properties for CCPR.

Property	Test Method	Requirement
Deleterious Materials: Clay Lumps and Friable Particles in Aggregate	AASHTO T 112 [48]	0.2% Maximum
Maximum Sieve Size, 1.5 inches (37.5 mm)	AASHTO T 27 [49]	100% passing

**Table 2:** RAP gradation requirement.

Sieve Size	Control Points	
	Min.	Max.
1/2”	100	100
3/4”	-	-
3/8”	-	-
No. 4	-	-
No. 200	2	9

**Table 3:** CCPR mix design criteria.

Test Method	Criteria
Foam-stabilized CCPR mix	
Moisture-Density Relations (AASHTO T180 [55], Method D)	Determined by design
Indirect Tensile Strength (ITS, AASHTO T283 [56])	Min. 45 psi (Dry) Min. 30 psi (Wet)
Retained Indirect Tensile Strength (AASHTO T283)	Min. 70% of Dry ITS Test Result
**Emulsion-stabilized CCPR mix**	
Moisture-Density Relations (AASHTO T180, Method D)	Determined by design
Marshall Stability Test (either ASTM 5581 [57] for 6 in. samples or AASHTO T245 [58] for 4 in. samples)	Min. 2500 lbs. for 6 in. diameter specimens Min. 1250 lbs. for 4 in. diameter specimens
Retained Stability (ASTM 5581 for 6 in. samples or AASHTO T 245 for 4 in. samples)	Min. 70% of Marshall Stability Test Result
Raveling Stability (ASTM D7196 [59])	Max. 2%
Thermal Cracking (Indirect Tensile Test, AASHTO T 322 [60])	Critical cracking temperature must be less than or equal to the pavement temperature given for the project climate area and pavement depth by LTPPBind

**Table 4:** CCPR structural layer coefficients in various studies.

Description	Layer Coefficient
Granular base	0.06–0.14
Indiana	0.22
New Hampshire	0.28
Quebec	0.30
Minnesota	0.35
Pennsylvania	0.37
Virginia	0.36–0.44
Conventional AC	0.35–0.54

## Data Availability

No new data were created or analyzed in this study. Data sharing is not applicable to this article.

## References

[1] Bemanian S., Polish P., Maurer G. (2006). Cold in-place recycling and full-depth reclamation projects by Nevada Department of Transportation: State of the practice. Transp. Res. Rec..

[2] Diefenderfer B.K., Bowers B.F., Schwartz C.W., Farzaneh A., Zhang Z. (2016). Dynamic modulus of recycled pavement mixtures. Transp. Res. Rec..

[3] Stroup-Gardiner M. (2021). Practice and Performance of Cold In-Place Recycling and Cold Central Plant Recycling.

[4] Diefenderfer B.K., Apeagyei A.K. (2014). I-81 In-Place Pavement Recycling Project.

[5] Al-Qadi I., Ozer H. (2020). In-Place and Central-Plant Recycling of Asphalt Pavements in Virginia.

[6] Stimilli A., Ferrotti G., Graziani A., Canestrari F. (2013). Performance evaluation of a cold-recycled mixture containing high percentage of reclaimed asphalt. Road Mater. Pavement Des..

[7] FHWA (2018). Overview of Project Selection Guidelines for Cold In-Place and Cold Central Plant Pavement Recycling. TechBrief.

[8] Apeagyei A.K., Diefenderfer B.K. (2013). Evaluation of cold in-place and cold central-plant recycling methods using laboratory testing of field-cored specimens. J. Mater. Civ. Eng..

[9] Amarh E.A., Santos J., Flintsch G.W., Diefenderfer B.K. (2022). Evaluating the Potential Environmental Benefits of Cold Recycling-Based Methods for Flexible Pavement Rehabilitation in Virginia. Transp. Res. Rec..

[10] Hansen K.R., Copeland A. (2016). Asphalt Pavement Industry Survey on Recycled Materials and Warm-Mix Asphalt Usage: 2015, 6th Annual Survey (IS138).

[11] Hansen K.R., Copeland A. (2017). Asphalt Pavement Industry Survey on Recycled Materials and Warm-Mix Asphalt Usage: 2016, 7th Annual Survey (IS138).

[12] Williams B.A., Copeland A., Ross T.C. (2018). Asphalt Pavement Industry Survey on Recycled Materials and Warm-Mix Asphalt Usage: 2017, 8th Annual Survey (IS 138).

[13] Williams B.A., Willis J.R., Shacat J. (2022). Asphalt Pavement Industry Survey on Recycled Materials and Warm-Mix Asphalt Usage: 2022, 13th Annual Survey (IS 138).

[14] Williams B.A., Willis J.R., Ross T.C. (2019). Asphalt Pavement Industry Survey on Recycled Materials and Warm-Mix Asphalt Usage: 2018, 9th Annual Survey (IS 138).

[15] Williams B.A., Willis J.R., Shacat J. (2021). Asphalt Pavement Industry Survey on Recycled Materials and Warm-Mix Asphalt Usage: 2020, 11th Annual Survey (IS 138).

[16] Ashtiani M.Z., Muench S.T., Gent D., Uhlmeyer J.S. (2019). Application of satellite imagery in estimating stockpiled reclaimed asphalt pavement (RAP) inventory: A Washington State case study. Constr. Build. Mater..

[17] Copeland A. (2011). Reclaimed Asphalt Pavement in Asphalt Mixtures: State of the Practice.

[18] Wielinski J., Zahrn T. (2021). Introduction to Cold Central Plant Recycling, 84th IAPA Annual Conference. https://www.il-asphalt.org/files/4616/1601/3632/2021WielinskiZahrn.pdf.

[19] Diefenderfer B.K., Boz I., Habbouche J., Jones D., Hand A.J., Bowers B.F., Flintsch G. (2021). Proposed AASHTO Practice and Tests for Process Control and Product Acceptance of Asphalt-Treated Cold Recycled Pavements.

[20] Gu F., Ma W., West R.C., Taylor A.J., Zhang Y. (2019). Structural performance and sustainability assessment of cold central-plant and in-place recycled asphalt pavements: A case study. J. Clean. Prod..

[21] Díaz-Sánchez M.A., Timm D.H., Diefenderfer B.K. (2016). Structural Assessment of the Effect of a Cement-Stabilized Base Combined with a Cold Central-Plant Recycled Layer at the NCAT Test Track. The Roles of Accelerated Pavement Testing in Pavement Sustainability.

[22] Magar S., Xiao F., Singh D., Showkat B. (2022). Applications of reclaimed asphalt pavement in India—A review. J. Clean. Prod..

[23] Diefenderfer B.K., Link S.D. Temperature and confinement effects on the stiffness of a cold central plant recycled mixture. Proceedings of the 12th International Society for Asphalt Pavements Conference on Asphalt Pavements.

[24] Bowers B.F., Gu F. (2021). Asphalt Pavement: A Critically Important Aspect of Infrastructure Resiliency. No. NCAT Report 21-02. https://eng.auburn.edu/research/centers/ncat/files/technical-reports/rep21-02.pdf.

[25] Flintsch G., Meijer J., Smith K. (2020). Improved Asphalt Sustainability Through Perpetual Pavement Design.

[26] Bowers B.F., Allain D.E., Diefenderfer B.K. (2020). Review of agency pavement recycling construction specifications. Transp. Res. Rec..

[27] Diefenderfer B.K., Bowers B.F., Apeagyei A.K. (2015). Initial Performance of Virginia’s Interstate 81 In-Place Pavement Recycling Project. Transp. Res. Rec..

[28] Xiao F., Yao S., Wang J., Li X., Amirkhanian S. (2018). A literature review on cold recycling technology of asphalt pavement. Constr. Build. Mater..

[29] Diefenderfer B.K., Timm D.H., Boz I., Bowers B.F. (2021). Structural Study of Cold Central Plant Recycling Sections at the National Center for Asphalt Technology (NCAT) Test Track: Phase III.

[30] (2004). Wirtgen Cold Recycling Manual 2004.

[31] Wacker B., Kalantari M., Diekmann M. (2020). Cold recycling in Germany—Current experiences and future projects. Proceedings of the 9th International Conference on Maintenance and Rehabilitation of Pavements—Mairepav9.

[32] Charmot S., Teh S.Y., Haris R.E.A., Ayob M.A., Ramzi M.R., Kamal D.D.M., Atan A. (2022). Field performance of bitumen emulsion Cold Central Plant Recycling (CCPR) mixture with same day and delayed overlay compared with traditional rehabilitation procedures. Case Stud. Constr. Mater..

[33] Cheng H., Sun L., Liu L., Li H. (2018). Fatigue characteristics of in-service cold recycling mixture with asphalt emulsion and HMA mixture. Constr. Build. Mater..

[34] Cox B.C., Howard I.L. (2013). Cold In-Place Recycling and Full Depth Reclamation Literature Review.

[35] Bowers B.F., Powell R.B. (2021). Use of a Hot-Mix Asphalt Plant to Produce a Cold Central Plant Recycled Mix: Production Method and Performance. Transp. Res. Rec..

[36] Diefenderfer B.K., Flintsch G., Xue W., Meroni F., Boz I., Timm D. (2022). Structural Performance of an Asphalt Pavement Containing Cold Central Plant Recycling and Full-Depth Reclamation. Transp. Res. Rec..

[37] Diefenderfer B.K., Diaz-Sanchez M., Timm D.H., Bowers B.F. (2017). Structural Study of Cold Central Plant Recycling Sections at the National Center for Asphalt Technology (NCAT) Test Track.

[38] Allain D., Bowers B.F., Vargas-Nordcbeck A., Lynn T. (2022). Pavement Recycling in Cold Climates: Laboratory and Field Performance of the MnROAD Cold Recycling and Full Depth Reclamation Experiment. Transp. Res. Rec..

[39] El-shaib M.A., El-Badawy S.M., Shawaly E.S.A. (2017). Comparison of AASHTO 1993 and MEPDG considering the Egyptian climatic conditions. Innov. Infrastruct. Solut..

[40] Ahmed Kazmi S., Bowers B.F., Diefenderfer B.K. (2021). Cold Central Plant Asphalt Mixture Stockpiling: Laboratory Evaluation. J. Mater. Civ. Eng..

[41] Wirtgen Group Cold Recycling on Germany’s Oldest Autobahn. https://lectura.press/en/article/cold-recycling-on-germany-s-oldest-autobahn/63784.

[42] Asphalt Recycling and Reclaiming Association (2017). CR102—Recommended Construction Guidelines for Cold Central Plant Recycling (CCPR) Using Bituminous Recycling Agents. https://www.arra.org/page/guideline.

[43] Diefenderfer B.K., Apeagyei A.K., Gallo A.A., Dougald L.E., Weaver C.B. (2012). In-Place Pavement Recycling on I-81 in Virginia. Transp. Res. Rec..

[44] Weaver C.B., Clark T. (2007). I-81 Southbound Lanes Pavement Recommendation from Exit 219 to 1.31 Miles North of Rockbridge County Line (MP 13.13 to MP 1.31). Augusta County. Memorandum.

[45] (2019). Standard Specifications for Road and Bridge Construction.

[46] (2016). Bituminous Mixtures—Material Specifications Part 8: Reclaimed Asphalt.

[47] Bowers B.F., Diefenderfer B.K., Cross S.A., Vargas A., Gu F. (2023). Construction Guidelines for Cold Central Plant Recycling and Cold In-Place Recycling.

[48] (2010). Standard Method of Test for Clay Lumps and Friable Particles in Aggregate.

[49] (2005). Sieve Analysis of Fine and Coarse Aggregate, Standard Specifications for Transportation Materials and Methods of Sampling and Testing.

[50] Li J., Uhlmeyer J.S., Mahoney J.P., Muench S.T. (2010). Updating the Pavement Design Catalogue for the Washington State Department of Transportation: Using 1993 AASHTO Guide, Mechanistic–Empirical Pavement Design Guide, and Historical Performance. Transp. Res. Rec..

[51] Khosravifar S., Schwartz C.W., Goulias D.G. (2013). Mechanistic structural properties of foamed asphalt stabilized base materials. Proceedings 92nd Annual Meeting of the Transportation Research Board.

[52] Santos J., Flintsch G., Ferreira A. (2017). Environmental and economic assessment of pavement construction and management practices for enhancing pavement sustainability. Resour. Conserv. Recycl..

[53] (2020). Standard Practice for Emulsified Asphalt Content of Cold Recycled Mixture Designs.

[54] (2015). Basic Asphalt Recycling Manual. Research Report FHWA-HIF-14-001.

[55] (2020). Standard Method of Test for Moisture–Density Relations of Soils Using a 4.54-kg (10-lb) Rammer and a 457-mm (18-in.) Drop.

[56] (2020). Standard Method of Test for Resistance of Compacted Asphalt Mixtures to Moisture-Induced Damage.

[57] (2025). Standard Test Method for Resistance to Plastic Flow of Asphalt (Bituminous) Mixtures Using Marshall Apparatus (6 in. Diameter Specimen).

[58] (2022). Standard Method of Test for Resistance to Plastic Flow of Asphalt Mixtures Using Marshall Apparatus.

[59] (2023). Standard Test Method for Raveling Test of Cold-Mixed Emulsified Asphalt Samples.

[60] (2007). Standard Method of Test for Determining the Creep Compliance and Strength of Hot-Mix Asphalt (HMA) Using the Indirect Tensile Test Device.

[61] (2020). Mechanistic-Empirical Pavement Design Guide: A Manual of Practice.

[62] (1993). Guide for Design of Pavement Structures.

[63] Loizos A. (2007). In-situ characterization of foamed bitumen treated layer mixes for heavy-duty pavements. Int. J. Pavement Eng..

[64] Masad E., Jandhyala V.K., Dasgupta N., Somadevan, Shashidhar N. (2002). Characterization of air void distribution in asphalt mixes using X-ray computed tomography. J. Mater. Civ. Eng..

[65] Mohammad L.N., Elseifi M.A., Cooper S.B., Raghavendra A. (2012). Evaluating Effects of Volumetric and Mechanistic Test Variability on Predicted Performance of Asphalt Pavement: Applying the Mechanistic–Empirical Pavement Design Guide. Transp. Res. Rec..

[66] Cosenza N., Robinson Z. Cold Central Plant—A View into Our Future. Proceedings of the Semi-Annual ARRA Conference.

[67] Schwartz C.W., Diefenderfer B.K., Bowers B.F. (2017). Material Properties of Cold-in-place Recycled and Full-depth Reclamation Asphalt Concrete. National Cooperative Highway Research Program Report 863.

[68] Dave E.V., Sias J.E., Nemati R. (2019). Layer Coefficients for New Hampshire Department of Transportation Pavement Design.

[69] Tarefder R.A., Bateman D. (2012). Design of optimal perpetual pavement structure. J. Transp. Eng..

[70] Tarsi G., Tataranni P., Sangiorgi C. (2020). The challenges of using reclaimed asphalt pavement for new asphalt mixtures: A review. Materials.

[71] (2022). INDOT Standard Specifications. Indianapolis, IN, USA. https://www.in.gov/dot/div/contracts/standards/book/index.html.

[72] Indiana Department of Transportation (INDOT) (2019). State Route 101 Success Story. https://www.in.gov/indot/engineering/files/SR-101-success_story.pdf.

[73] Timm D.H., Diefenderfer B.K., Bowers B.F., Flintsch G. (2021). Utilization of Cold Central Plant Recycled Asphalt in Long-Life Flexible Pavements. Transp. Res. Rec..

[74] (2021). Fresno County Success Story. https://roadresource.org/resources/125/story.pdf.

[75] Virginia Department of Transportation, Materials Division (2017). Pavement ME User Manual—Draft, Richmond. https://www.vdot.virginia.gov/media/vdotvirginiagov/doing-business/tools/geotechnical/asset_upload_file108_3638.pdf.

[76] Virginia Department of Transportation (2015). Special Provision for Cold Central Plant Recycling Material Placement, Richmond. https://www.vdot.virginia.gov/media/vdotvirginiagov/doing-business/technical-guidance-and-support/technical-guidance-documents/construction/migrated-acc/07RevDiv_III_acc071822.pdf.

[77] Willis J.R., Timm D.H. (2010). Development of Stochastic Perpetual Pavement Design Criteria. J. Assoc. Asph. Paving Technol..

[78] Díaz-Sánchez M.A., Timm D.H., Diefenderfer B.K. (2017). Structural coefficients of cold central plant recycled asphalt mixtures. J. Transp. Eng. Part A Syst..

[79] Timm D.H., Diefenderfer B.K., Bowers B.F. (2018). Cold central plant recycled asphalt pavements in high traffic applications. Transp. Res. Rec..

[80] Tompkins D., Zammarchi M., Rettner D.L. (2021). Cold Central Plant Recycling (CCPR)–National Road Research Alliance (NRRA).

[81] Bhatt B., Wu S. (2025). A comprehensive state-of-art review on the use of rejuvenators in asphalt pavement. J. Road Eng..

